# Collision Prediction and Social-Norm-Fusion-Based Social-Navigation Method for Quadruped Robots

**DOI:** 10.3390/biomimetics11040228

**Published:** 2026-03-31

**Authors:** Junxian Bei, Qingyun Zhu, Zhuorong Shi, Yonghua Liu

**Affiliations:** School of Automation, Guangdong University of Technology, Guangzhou 510006, China; superbei11@126.com (J.B.); zhuqingyun_1999@163.com (Q.Z.); as1033813371@gmail.com (Z.S.)

**Keywords:** quadruped robots, social navigation, social force model, dynamic obstacle avoidance, path planning

## Abstract

As a typical biomimetic robotic system, quadruped robots replicate the flexible locomotion of quadruped mammals, outperforming wheeled robots in human-centered daily scenarios. To improve the social navigation adaptability of biomimetic quadruped robots in human–robot shared environments, this paper proposes a collision-aware orthogonal steering social force model (COSFM), an enhanced social force model that integrates collision prediction and social norms, inspired by human-like collision avoidance behaviors and social interaction rules. The model addresses key limitations of conventional social force models: delayed responses to dynamic pedestrians and inadequate consideration of pedestrians’ comfort zones. It introduces a time-to-collision prediction mechanism to mimic human predictive decision-making in dynamic social interactions, enhancing the robot’s anticipation of pedestrian motion intentions, and designs an orthogonal steering-based avoidance strategy for four typical human–robot interaction scenarios (head-on encounters, intersecting paths, active overtaking, passive yielding). This strategy replicates humans’ natural priority of lateral steering over abrupt deceleration or retreat, generating socially compliant trajectories aligned with human behavioral expectations. The proposed method is validated via simulation and real-world experiments on a Unitree Aliengo quadruped robot. Results show that the COSFM algorithm achieves a higher navigation success rate and better performance in path length, navigation time, and minimum human-robot distance than existing approaches, while its human-like lateral avoidance priority effectively preserves pedestrians’ psychological comfort zones, demonstrating robust social adaptability and great application potential for biomimetic legged robots.

## 1. Introduction

In recent years, with the rapid advancement of robotic technologies, mobile robots have expanded from traditional industrial applications to daily-life scenarios, including service guidance, logistics delivery, and social companionship, thanks to their flexibility and strong environmental adaptability [1,2]. In human-populated environments such as urban streets and shopping malls, robot navigation requires both accurate prediction of human motion behavior and effective integration of these predictions with efficient path planning to enable safe, natural, and socially acceptable human–robot coexistence. Current robot navigation research has developed two mainstream technical approaches: interpretable rule-based model-driven methods, represented by the social force model (SFM), and data-driven deep reinforcement learning (DRL) methods, both of which face core bottlenecks in dynamic human-robot shared environments.

Biomimetics, which designs engineering systems inspired by biological structures, locomotion mechanisms, and behavioral patterns, provides the core theoretical foundation for the development of legged robots and social navigation technology. From the perspective of locomotion biomimetics, quadruped robots replicate the musculoskeletal structure and dynamic gait patterns of quadruped mammals, with superior omnidirectional mobility, including flexible lateral movement, in-situ steering, and dynamic gait adjustment. This not only enables their adaptation to complex, unstructured daily scenarios but also provides a physical basis for natural human-like social interaction behaviors. Meanwhile, from a behavioral biomimetics perspective, the widely used SFM stems from the biomimetic modeling of pedestrian crowd motion dynamics, yet most existing SFM-based navigation methods ignore the alignment between bionic behavioral models and the bionic locomotion characteristics of quadruped robot platforms. This leads to a prominent gap between theoretical social navigation models and their stable deployment on physical legged robots, which is the core problem addressed in this work.

Conventional navigation methods primarily target static or predictable obstacles, whereas social navigation focuses on coordinating human-robot behaviors in shared spaces. Social force models are widely used in robot social navigation for their capability to explicitly model inter-agent interactions [3,4,5]. However, most existing approaches are designed for specific interaction tasks and mainly validated on wheeled platforms with limited mobility, restricting their maneuverability and social adaptability in complex crowd environments. Meanwhile, three key technical challenges remain unsolved in current social navigation research: the inherent delay in responding to dynamic pedestrians, insufficient consideration of pedestrians’ social comfort zones, and a lack of systematic adaptation to legged robot locomotion constraints. In contrast, quadruped robots have greater locomotion flexibility, including lateral motion, in-situ turning, and dynamic gait adjustment, enabling more natural and socially compliant obstacle avoidance [6,7]. Nevertheless, classical SFM relies on instantaneous state information and often shows delayed responses in dynamic interactions, with the above challenges further limiting its effectiveness when applied to quadruped robots.

Existing collision-prediction-based SFM improves proactive obstacle avoidance by estimating future collision risks; however, their avoidance forces are typically aligned with relative position or velocity directions, making robots rely mainly on deceleration, backward motion, or even stagnation, and thus failing to balance efficiency, motion continuity, and social compliance in dynamic human–robot interactions. Meanwhile, DRL, another dominant social navigation paradigm, enables end-to-end learning of complex human-robot interaction policies without handcrafted rules; however, it has inherent limitations, including poor interpretability, limited cross-scenario generalization, and significant sim-to-real gaps, which limit its reliable deployment in real-world safety-critical navigation tasks. These unresolved issues further exacerbate the aforementioned core challenges. To address this gap, this study proposes a collision-aware orthogonal steering social force model, which introduces a socially compliant orthogonal steering strategy (OSS) based on time-to-collision (TTC) prediction to reformulate the direction of avoidance forces. By encouraging lateral detouring while maintaining safe interpersonal distances, the proposed model achieves more natural and efficient avoidance behaviors. Furthermore, COSFM is extended by integrating quadruped-specific kinematic characteristics and gait constraints, enabling stable and reliable deployment on real quadruped robot platforms.

The main contributions of our work include the following:

- Within the SFM framework, collision prediction and orthogonal steering strategies are unified into a single formulation, improving behavioral responses in dynamic obstacle avoidance;
- For quadruped robot platforms, gait awareness, leg safety spaces, and dynamic balance constraints are systematically integrated to adapt social navigation models to legged locomotion;
- A rule-driven steering decision mechanism is designed for multiple typical human–robot interaction scenarios, improving the social compliance and predictability of navigation behaviors.

## 2. Related Work

To mitigate the inherent delay in classical SFM for dynamic obstacle avoidance, extensive studies have introduced collision prediction mechanisms to enable proactive navigation. Karamouzas et al. [8] first proposed a predictive collision avoidance model that estimates future collision risks to generate smooth trajectories for pedestrian simulation. Building on this, Zanlungo et al. [9] designed an explicit TTC that dynamically adjusts interaction forces based on predicted collision time and the minimum closest approach distance. Kivrak et al. [10,11] further combined TTC-based local planning with global path planning for proactive obstacle avoidance, yet their work has limited modeling of social cues and interaction conventions. Martini et al. [12] integrated the dynamic window approach with SFM and optimized model parameters via DRL, without systematically resolving the local minimum problem. Jafari et al. [13,14] adopted TTC-guided deceleration strategies to reduce collision risk, while frequent deceleration inevitably degrades navigation efficiency. These works only optimize the magnitude of interaction forces while retaining the repulsive force direction aligned with relative position/velocity, which cannot fundamentally solve the efficiency loss caused by over-reliance on deceleration and retreat behaviors.

Subsequent works further optimized SFM for social compliance across multiple dimensions. Bilen et al. [15] dynamically adjusted interaction distances based on pedestrians’ emotional states, but its reliance on affective perception limits real-world deployment reliability. Vega et al. [16] proposed an adaptive spatial density function for refined personal space modeling, while Patompak et al. [17] fused discrete social cues via fuzzy reasoning, both constrained by strict sensing requirements for engineering implementation. Reddy et al. [18] introduced orthogonal social forces to enforce social convention-compliant anticipatory turning, but lacked dynamic adaptation mechanisms. Ægidius et al. [19] proposed the augmented social force model, a legged robot-oriented SFM variant to improve robustness and social compliance; however, it is unable to isolate and verify the independent effectiveness of a single core innovation and exhibits limited generalization performance in unstructured dynamic pedestrian scenarios. Boldrer et al. [20] combined potential fields with elliptical limit cycles to generate smooth, socially aware real-time trajectories, while adaptability to non-cooperative obstacles and parameter robustness remain open issues. Existing orthogonal force-based methods lack a rule-driven steering decision mechanism for typical human-robot interaction scenarios, leading to poor predictability and generalization of socially compliant behaviors.

Beyond rule-based SFM, DRL has emerged as a dominant paradigm for social navigation, enabling end-to-end learning of complex human-robot interaction policies without handcrafted rules. Chen et al. [21] proposed the collision avoidance with DRL (CADRL) algorithm, which employs a value network to map agent states to navigation actions, generating collision-free trajectories by maximizing one-step lookahead values for decentralized multi-agent avoidance. Chen et al. [22] extended this to pedestrian-dense scenarios with the socially aware collision avoidance with DRL (SA-CADRL) algorithm, embedding social norms into the reward function to enhance the social compliance of learned behaviors. Han et al. [23] integrated reciprocal velocity obstacle theory into DRL, shaping the state space and rewards to facilitate reciprocal collision avoidance in distributed multi-robot systems. Additionally, Cui et al. [24] addressed safety by designing a control barrier function to constrain and optimize policy network outputs, providing certifiable safety guarantees for dynamic navigation tasks. Despite these advances, DRL-based methods have inherent limitations: they achieve a minimum human-robot separation of only 0.117–0.199 m [21,22], far below the 0.5 m social comfort threshold, and suffer from poor interpretability and a significant sim-to-real gap.

Despite extensive advances in collision-prediction-enhanced SFM, social-compliance-optimized SFM, and DRL-based social navigation, enabling natural human-robot coexistence for quadruped robots in real-world mixed environments remains an unresolved challenge. Existing predictive SFM variants only optimize interaction force magnitude without reforming repulsive force direction, causing unavoidable navigation efficiency loss from over-reliance on deceleration; existing social compliance-focused SFM lacks scenario-based rule-driven steering decision mechanisms, with poor generalization of socially compliant behaviors; and DRL methods suffer from inherent defects, including weak interpretability, poor efficiency, significant sim-to-real gaps, and insufficient consideration of quadruped-specific motion constraints. These core bottlenecks in existing methods are the primary motivation for this work.

## 3. Collision-Aware Orthogonal Steering Social Force Model

### 3.1. Classical Social Force Model

The SFM, originally proposed by Helbing in 1995 [25], is a mathematical framework grounded in physical principles for describing the motion behavior of pedestrians or crowds. By quantitatively modeling the driving force, social interaction forces, and physical constraint forces acting on individuals during motion, the model captures the interaction between individual behaviors and the surrounding environment. Due to its interpretability and computational efficiency, the SFM has been widely applied in crowd evacuation simulation, public space design optimization, and pedestrian behavior prediction.

**Figure 1:**
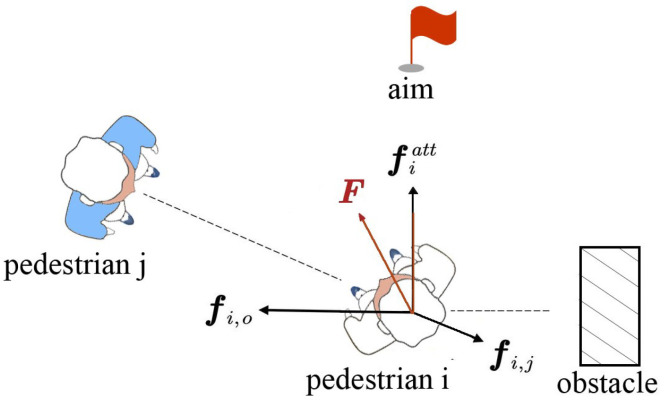
Illustration of the classical social force model for robot–pedestrian interaction.

As illustrated in Figure 1, pedestrian *i* moves under the action of a resultant force $F_{i}$, which is composed of three components: The goal-directed driving force $f_{i}^{att}$, the pedestrian interaction force $f_{i,j}$, and the obstacle-induced force $f_{i,o}$. The goal-directed driving force guides the pedestrian toward the target position and reflects a tendency to move in the desired direction at a preferred speed. This force is jointly determined by the desired velocity and the current velocity, and can be expressed as:

(1) $$f_{i}^{att}=\frac{v_{i}^{d}e_{i}^{d}-v_{i}\left(t\right)}{\tau_{i}}$$

where $v_{i}\left(t\right)$ denotes the actual velocity of pedestrian *i* at time *t*; $\tau_{i}$ is the relaxation time; $v_{i}^{d}$ represents the magnitude of the desired speed; and $e_{i}^{d}$ is the unit vector indicating the desired moving direction, which can be expressed as:

(2) $$e_{i}^{d}=\frac{r_{d}-r_{i}\left(t\right)}{∥r_{d}-r_{i}\left(t\right)∥}$$

In addition to the goal-directed driving force, pedestrian motion is also influenced by surrounding pedestrians, which is typically modeled as an exponentially decaying repulsive force with respect to inter-personal distance:

(3) $$f_{i,j}=Ae^{\frac{\left(R_{i}+R_{j}-∥d_{i,j}∥\right)}{B}}\frac{-d_{i,j}}{∥d_{i,j}∥}$$

where *A* and *B* are parameters controlling the interaction strength and effective range of the force, respectively; $R_{i}$ and $R_{j}$ denote the radii of pedestrians *i* and *j*; and $d_{i,j}$ represents the distance vector between their centers of mass, defined as $d_{i,j}=r_{j}-r_{i}$.

Similarly, the repulsive force exerted by obstacle *o* on pedestrian *i* can be written as:

(4) $$f_{i,o}=A_{o}e^{\frac{\left(R_{i}-∥d_{i,o}∥\right)}{B_{o}}}\frac{-d_{i,o}}{∥d_{i,o}∥}$$

Considering that pedestrians have stronger perception in the forward direction and weaker perception behind them, an anisotropic factor is introduced to quantify this directional sensitivity difference:

(5) $$\omega =\lambda_{i}+\left(1-\lambda_{i}\right)\frac{1+\cos\varphi }{2}$$

where φ denotes the angle between the velocity vector $v_{i}$ and the distance vector $d_{i,j}$ (or $d_{i,o}$), and the parameter $\lambda_{i}\in \left[0,1\right]$ characterizes the sensitivity difference of pedestrian *i* to stimuli from different directions.

Based on Newton’s second law, the motion equation of pedestrian *i* can be expressed as:

(6) $$m_{i}\frac{dv_{i}}{dt}=f_{i}^{att}+\sum_{j\neq i}\omega_{i,j}f_{i,j}+\sum_{o}\omega_{i,o}f_{i,o}$$

### 3.2. Collision-Aware Social Force with Time-to-Collision

**Figure 2:**
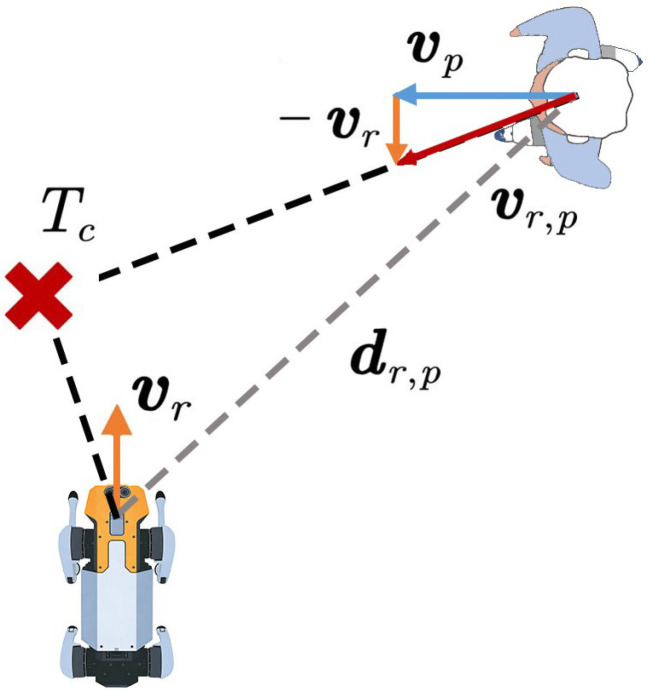
Definition and a geometric illustration of time-to-collision.

In the classical SFM, repulsive forces are computed solely based on the relative positions between the robot and pedestrians, while the influence of motion states on future collision risks is neglected. To address this issue, this study introduces the concept of time to collision $T_{c}$ proposed by Zanlungo et al. [9]. As illustrated in Figure 2, $T_{c}$ is defined as:

(7) $$T_{c}=-\frac{d_{r,p}\cdot v_{r,p}}{\|v_{r,p}{\|}^{2}}$$

where $r_{p}$ and $r_{r}$ denote the position vectors of the pedestrian and the robot, respectively; $v_{p}$ and $v_{r}$ denote their corresponding velocity vectors; $d_{r,p}=r_{p}-r_{r}$ represents the distance vector between the pedestrian and the robot; and $v_{r,p}=v_{p}-v_{r}$ denotes the relative velocity vector between them.

**Figure 3:**
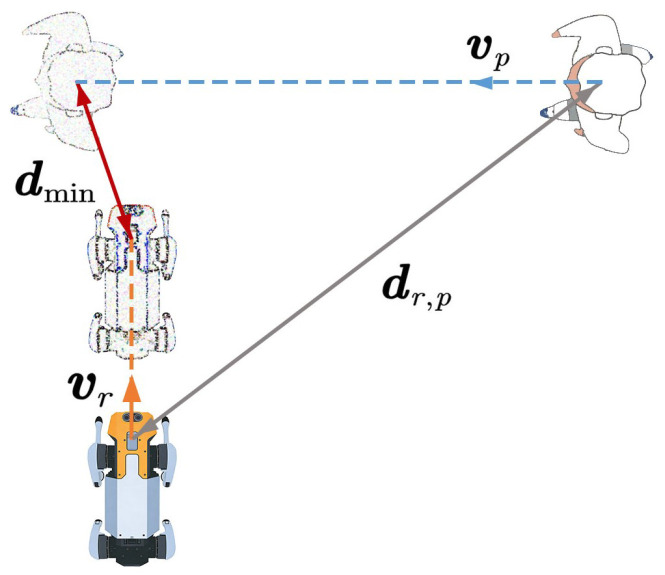
Geometric illustration of the minimum potential distance at closest approach.

Equation (7) predicts the time to collision between the robot and a pedestrian or an obstacle by computing the projection of the distance vector $d_{r,p}$ onto the relative velocity vector $v_{r,p}$. It should be noted that the predicted collision time $T_{c}$ does not represent the actual collision time; instead, it denotes the time required to reach the point of closest approach under the assumption that both agents maintain their current velocities and move along straight-line trajectories. If the distance between the two agents at this closest point exceeds a predefined safety distance threshold, a collision is considered unlikely to occur. As illustrated in Figure 3, the minimum potential distance $d_{min}$ can be used to characterize the separation at the point of closest approach and is given by:

(8) $$d_{min}=\left|\right|r_{p}^{pred}-r_{r}^{pred}\left|\right|$$

where $r_{p}^{pred}$ and $r_{r}^{pred}$ denote the predicted positions of the pedestrian and the robot after time $T_{c}$, respectively, which are computed as follows:

(9) $$\begin{array}{cc} r_{p}^{pred} & =r_{p}+v_{p}T_{c} \end{array}$$

(10) $$\begin{array}{cc} r_{r}^{pred} & =r_{r}+v_{r}T_{c} \end{array}$$

By incorporating the time-to-collision into the pedestrian repulsive force, the robot can dynamically choose between decelerating to yield and accelerating to pass based on the current distance to the pedestrian and the predicted collision time. The pedestrian repulsive force augmented with collision prediction can be expressed as:

(11) $$f_{r,p}^{cp}=A_{cp}e^{\left(-\frac{T_{c}}{B_{cp}}\right)}\left(-\frac{d_{r,p}}{∥d_{r,p}∥}\right)$$

where $A_{cp}$ is the amplitude coefficient of the steering force, which controls the force magnitude, and $B_{cp}$ is the decay coefficient that regulates the rate at which the force attenuates with distance.

### 3.3. Orthogonal Steering Strategy

Parisi et al. [26] analyzed pedestrian conflicts under different traffic flows and crowd sizes and found that, among the two primary collision avoidance behaviors—steering and deceleration—pedestrians tend to avoid collisions by changing their moving direction rather than by abrupt deceleration or stopping. For robots, repulsive forces acting directly in the backward direction may lead to unnatural retreating behaviors, which not only reduce navigation efficiency but may also cause confusion or discomfort to nearby pedestrians. Therefore, when avoiding pedestrians, steering behaviors should be prioritized, with deceleration adopted only when steering is constrained.

To better reproduce human-like avoidance patterns, an orthogonal steering strategy is introduced on the basis of Equation (11). Accordingly, a collision prediction-based orthogonal steering interaction force is formulated as follows:

(12) $$f_{r,p}^{cp}=A_{cp}e^{\left(-\frac{T_{c}}{B_{cp}}\right)}R\left(\theta \right)\left(-\frac{d_{r,p}}{∥d_{r,p}∥}\right)$$

where θ denotes the rotation angle of the force, which is selected as π/2 or −π/2 according to the chosen avoidance direction. R(θ) represents the rotation matrix used to change the force direction, which is expressed as:

(13) $$R\left(\theta \right)=\left(\begin{array}{cc} \cos\theta & -\sin\theta \\ \sin\theta & \cos\theta \end{array}\right)$$

**Figure 4:**
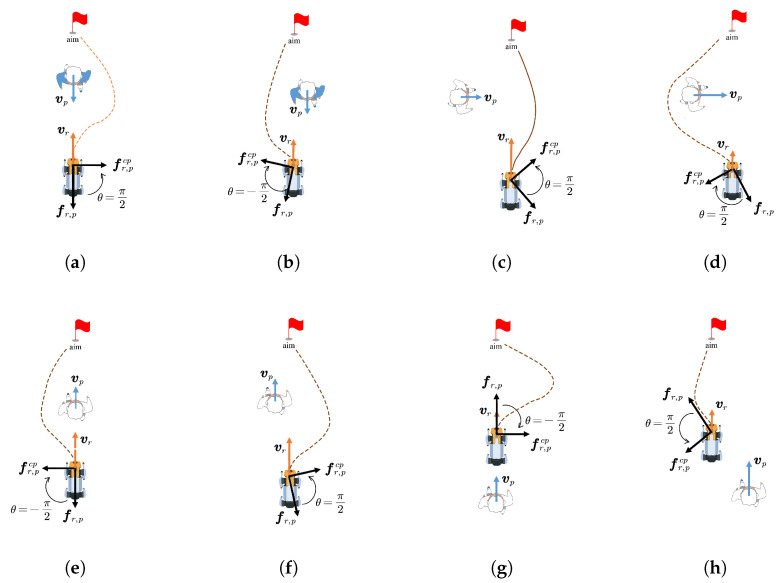
Illustration of orthogonal steering directions in four typical human–robot interaction scenarios. (**a**) Scenario 1, right evasion. (**b**) Scenario 1, left evasion. (**c**) Scenario 2, right evasion. (**d**) Scenario 2, left evasion. (**e**) Scenario 3, right evasion. (**f**) Scenario 3, left evasion. (**g**) Scenario 4, right evasion. (**h**) Scenario 4, left evasion.

In dynamic human–robot shared environments, the selection of avoidance direction is inherently scenario-dependent and must jointly consider real-time pedestrian states and social behavioral norms. To this end, we analyze four typical human–robot interaction scenarios (head-on encounters, intersecting paths, active overtaking, and passive yielding, as illustrated in Figure 4), construct a rule-driven steering model grounded in social navigation conventions to generate avoidance behaviors aligned with human expectations, and clarify the corresponding steering decision rules for each scenario: prioritizing right-side avoidance for head-on encounters, dynamically choosing between proactive passing and yielding for intersecting paths, following traffic norms for left-side priority overtaking, and proactively creating a compliant overtaking corridor for passive yielding. On this basis, we define β∈[0,2π) as the minimum counterclockwise rotation angle required for collinear alignment of two vectors, formulate a unified steering decision criterion for avoidance direction selection, and derive the general computational framework for the steering angle θ.

(14) $$\theta =\left\{\begin{array}{cc} \frac{\pi }{2} & \beta <\pi \\ -\frac{\pi }{2} & \beta >\pi \end{array}\right.$$

When β=π, the steering strategy is determined by the directional relationship of velocities:

(15) $${\theta |}_{\beta =\pi }=\left\{\begin{array}{cc} -\frac{\pi }{2} & v_{r}\cdot v_{p}\geq 0\\ \frac{\pi }{2} & v_{r}\cdot v_{p}<0 \end{array}\right.$$

## 4. Quadruped Robot Adaptation and Navigation Pipeline

### 4.1. Quadruped Robot Adaptation

In view of the unique kinematic characteristics and biological motion patterns of quadruped robots, this subsection introduces four key optimizations into the COSFM framework to ensure that the robot’s navigation behavior fully adapts to its physical structure and motion constraints while complying with social norms.

#### 4.1.1. Motion Mode Constraints and Steering Mapping

Although quadruped robots possess omnidirectional mobility, this paper introduces biomimetic motion mode constraints to enhance the naturalness of their locomotion behavior and improve pedestrian acceptance. Referring to the locomotion characteristics of real canines [27], quadruped robots should prioritize obstacle avoidance through yaw steering rather than direct lateral movement during regular navigation. Specifically, let the resultant force generated by the COSFM be $F_{total}$ and the current heading angle of the robot be ψ, its forward unit vector and normal unit vector are

(16) $$e_{h}={\left[\begin{array}{cc} \cos\psi & \sin\psi \end{array}\right]}^{⊤}$$

(17) $$e_{n}={\left[\begin{array}{cc} -\sin\psi & \cos\psi \end{array}\right]}^{⊤}$$

Then the social resultant force can be decomposed into a forward component along the heading direction and a lateral component perpendicular to the heading direction:

(18) $$\begin{array}{c} F_{\|}=F_{total}\cdot e_{h} \end{array}$$

(19) $$\begin{array}{c} F_{⊥}=F_{total}\cdot e_{n} \end{array}$$

Under this motion pattern constraint, the lateral component $F_{⊥}$ is no longer directly mapped to a lateral velocity command. Instead, it is used to generate a desired yaw angular velocity, such that lateral avoidance is achieved through heading adjustment:

(20) $${\dot{\psi }}_{d}=k_{\psi }\tanh\;\left(\frac{F_{⊥}}{F_{0}}\right)$$

where $k_{\psi }$ denotes the steering sensitivity coefficient, and $F_{0}$ is a normalization constant introduced to suppress high-frequency steering caused by small lateral disturbances.

To handle extreme close-range interactions or sudden collision risks, an urgency metric based on the predicted TTC $T_{C}$ is introduced:

(21) $$\kappa =\frac{1}{T_{c}}$$

When κ exceeds a predefined threshold $\kappa_{crit}$ (set to $0.5\;s^{-1}$ in this work, with an empirical range of 0.3–$0.7\;s^{-1}$, corresponding to a critical predicted collision time $T_{c}^{*}$ of about 1.4 s to 3.3 s), the robot is allowed to activate a constrained lateral translation capability as a supplementary avoidance strategy. The corresponding lateral velocity is defined as:

(22) $$v_{y,des}=\left\{\begin{array}{cc} 0, & \kappa <\kappa_{crit},\\ \mathrm{clip}\left(k_{y}F_{⊥},\;-v_{y,max},\;v_{y,max}\right), & \kappa \geq \kappa_{crit}. \end{array}\right.$$

where $v_{y,des}$ represents the maximum allowable lateral velocity and $k_{y}$ is a proportional gain used to regulate the sensitivity of lateral velocity to the interaction force.

Through the above constraint mechanisms, lateral social interaction forces are preferentially transformed into heading adjustment behaviors. As a result, the quadruped robot exhibits more natural, smooth, and socially intuitive avoidance trajectories in most human–robot interaction scenarios.

#### 4.1.2. Gait-Aware Collision Prediction

The gait cycle characteristics of quadruped robots have a significant influence on their dynamic response. During the swing phase, the reduction in ground contact points leads to decreased stability and increased prediction uncertainty; in contrast, during the stance phase, the robot exhibits stronger maneuverability. To account for this effect, the computation of the TTC $T_{c}$ is synchronously modified according to the gait cycle:

(23) $$T_{c}^{*}=T_{c}+\Delta t_{gait}$$

where $\Delta t_{gait}$ is the gait phase offset, defined as

(24) $$\Delta t_{gait}=\mu \cdot \left(1-\phi_{norm}\right)$$

where $\phi_{norm}\in \left[0,1\right]$ is the normalized gait phase (0 indicates the start of the stance phase, 1 indicates the end of the swing phase), and β is the uncertainty coefficient.

When the robot is in the swing phase, the predicted collision time is appropriately extended so that it initiates the avoidance action earlier to compensate for the response delay caused by the decrease in stability. This mechanism effectively reduces the discontinuity of avoidance caused by the gait cycle and makes the navigation trajectory smoother.

#### 4.1.3. Leg Safety Space Expansion

The traditional safety distance $d_{min}$ only considers the minimum distance between the robot’s torso and pedestrians, ignoring the leg swing space of quadruped robots. To avoid collisions between the robot’s feet and pedestrians, this paper extends the safety distance to

(25) $$d_{min}^{*}=d_{min}+\gamma \cdot l_{leg}\cdot \cos\left(\theta_{swing}\right)$$

where $l_{leg}$ is the leg length, $\theta_{swing}$ is the current leg swing angle, and γ is the safety coefficient. When the leg is fully extended, the safety distance increases by about 0.25 m, thus effectively avoiding collisions between the robot’s legs and pedestrians’ feet.

#### 4.1.4. Dynamic Balance Constraints

In the social navigation process based on the COSFM, the robot often needs to perform rapid steering or lateral adjustment actions when avoiding pedestrians, which will inevitably introduce additional lateral acceleration and angular acceleration. For quadruped robots, an excessively large instantaneous acceleration may cause the Zero Moment Point (ZMP) [28] to deviate from the support polygon, thus destroying dynamic stability. Therefore, it is necessary to impose dynamic balance constraints on the motion commands generated during the avoidance process while maintaining the rationality of social behavior. Assuming that the height of the robot’s center of mass is h, under the condition of walking on a flat ground, the relationship between its lateral acceleration and the lateral offset of the ZMP $d_{ZMP}$ approximately satisfies:

(26) $$d_{ZMP}=\frac{h}{g}\;\left|a_{y}\right|$$

To ensure the dynamic stability of the robot during avoidance, the ZMP is required to be always inside the current support polygon. Let $d_{safe}$ denote the minimum safe distance from the ZMP to the boundary of the support polygon, then the lateral acceleration must satisfy the following constraint:

(27) $$|a_{y}|\leq \frac{g}{h}\;d_{safe}$$

Under the condition that forward motion is dominant and lateral slip is negligible, the lateral acceleration of the center of mass is mainly caused by steering motion. According to the planar rigid body kinematics relationship, the lateral acceleration can be approximately expressed as:

(28) $$a_{y}\approx v_{x}\;\dot{\psi }$$

Accordingly, the stability constraint on the yaw angular velocity can be derived as:

(29) $$|\dot{\psi }|\leq \frac{g}{h}\;\frac{d_{safe}}{|v_{x}|}$$

In actual implementation, the above constraint is used as a clipping condition for the desired yaw angular velocity ${\dot{\psi }}_{d}$ generated by the COSFM. When the predicted steering action may cause the ZMP to cross the boundary, it is limited in amplitude without changing the avoidance direction itself. This strategy effectively avoids the dynamic instability problem caused by aggressive steering on the premise of not destroying the rationality of social avoidance decisions.

The ZMP approximation $d_{\mathrm{ZMP}}=\left(h/g\right)\cdot \left|a_{y}\right|$ is valid for quasi-static motion with $|a_{y}|<0.3g$, which matches the smooth steering requirements of social navigation. For highly dynamic scenarios with larger lateral accelerations, this approximation may deviate, which is a limitation to be addressed in future work.

In summary, by incorporating the above four optimization strategies into the original COSFM framework, this study establishes a navigation system that jointly considers social compliance and the physical maneuverability of quadruped robots. These enhancements transform the COSFM defined in Section 3 into a practical algorithm that is suitable for and deployable on real quadruped robot platforms. The pseudocode of the proposed COSFM quadruped robot navigation algorithm is presented in Algorithm 1.

**Algorithm 1** Pseudocode of COSFM1Input: $r_{r}$, $v_{r}$, $r_{p}$, $v_{p}$, $d_{safe}$, $\kappa_{crit}$, $\phi_{norm}$, $A_{cp}$, $B_{cp}$2$d_{r,p}\leftarrow r_{p}-r_{r}$; $v_{r,p}\leftarrow v_{p}-v_{r}$3$T_{c}\leftarrow -\frac{d_{r,p}\cdot v_{r,p}}{∥v_{r,p}{∥}^{2}}$; $d_{min}\leftarrow ∥\left(r_{p}+v_{p}T_{c}\right)-\left(r_{r}+v_{r}T_{c}\right)∥$4$T_{c}^{*}\leftarrow T_{c}+\mu \cdot \left(1-\phi_{norm}\right)$; $d_{min}^{*}\leftarrow d_{min}+\gamma l_{leg}cos\left(\theta_{swing}\right)$; $\kappa \leftarrow 1/T_{c}^{*}$5**if** 
$d_{min}^{*}<d_{safe}$ 
**then**
6   $\beta \leftarrow ∠(d_{r,p},v_{r,p})$7   **if** β=π **then**8      θ←π/2 **if** $v_{r}\cdot v_{p}\geq 0$ **else** −π/29   **else if** β∈[0,π/2)∪[3π/2,2π) **then** θ←π/210   **else** θ←−π/211   $R\left(\theta \right)\leftarrow \left[\begin{array}{cc} \cos\theta & -\sin\theta \\ \sin\theta & \cos\theta \end{array}\right]$12   $f_{cp}\leftarrow A_{cp}\exp\left(-\frac{T_{c}^{*}}{B_{cp}}\right)R\left(\theta \right)\frac{-d_{r,p}}{∥d_{r,p}∥}$13
**else**
14   $f_{cp}\leftarrow 0$15
**end if**
16

$F_{total}\leftarrow f_{i}^{att}+\sum_{j\neq i}\omega_{i,j}f_{i,j}+\sum_{o}\omega_{i,o}f_{i,o}+f_{\mathrm{cp}}$

17$e_{n}\leftarrow {\left[\begin{array}{cc} -\sin\psi & \cos\psi \end{array}\right]}^{⊤}$; $F_{⊥}\leftarrow F_{total}\cdot e_{n}$18

${\dot{\psi }}_{d}\leftarrow k_{\psi }\tanh\;\left(\frac{F_{⊥}}{F_{0}}\right)$

19

${\dot{\psi }}_{cmd}\leftarrow \mathrm{clip}\left({\dot{\psi }}_{d},{\dot{\psi }}_{ZMP}^{min},{\dot{\psi }}_{ZMP}^{max}\right)$

20

$a_{y}\leftarrow v_{x}{\dot{\psi }}_{d}$

21**if** 
$\kappa >\kappa_{crit}$ 
**then** 
$a_{cmd}\leftarrow \left[0,a_{y}\right]$ 
**else** 
$a_{cmd}\leftarrow \left[0,0\right]$
22Output: $v_{cmd}\leftarrow a_{cmd}$

### 4.2. Navigation Pipeline

**Figure 5:**
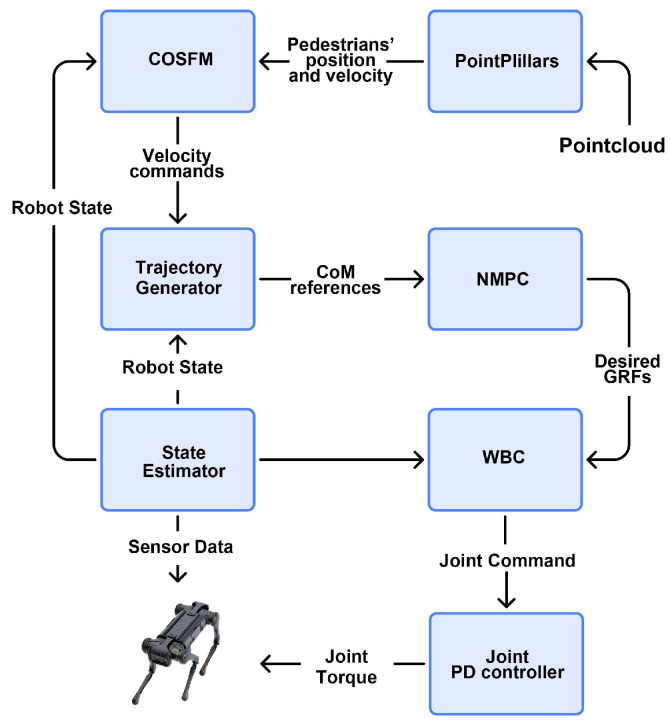
Overall pipeline of the proposed COSFM social navigation for quadruped robots.

The resultant force generated by the COSFM depicts the desired motion trend of the robot in the social space, which can be equivalently regarded as the desired acceleration at the centroid level. The trajectory generator performs numerical integration on this desired acceleration within a limited planning horizon to generate a smooth desired centroid position and velocity trajectory, which serves as the tracking reference input of the nonlinear model predictive control (NMPC) [29] module. Built on a standard optimal control framework, the NMPC module takes the robot’s real-time state feedback, full-body rigid dynamics, and contact/friction cone constraints as additional inputs and outputs the optimized centroid motion trajectory, foot contact forces, and joint reference sequence via receding-horizon optimization. The whole-body control (WBC) [30] module then takes these multi-task references from NMPC as the core input, solves the weighted quadratic programming problem under full-body dynamics and actuation constraints, and outputs feedforward joint torque commands. Finally, combined with joint-layer PD feedback control, the joint execution commands are generated to drive the robot, enabling it to maintain dynamic stability while strictly complying with social navigation norms. Hereby, the quadruped robot can be driven to perform pedestrian collision avoidance during navigation, given the availability of the position and velocity information of surrounding pedestrians. As the focus of this work is placed on the navigation methodology, the detailed motion control approaches for quadruped robots are not elaborated in this paper, and interested readers are referred to the relevant references for further information. The complete COSFM social navigation process of the quadruped robot is shown in Figure 5, where CoM indicates the center of mass and GRFs indicate ground reaction forces.

## 5. Experiments

### 5.1. Quadruped Robot Platform

**Figure 6:**
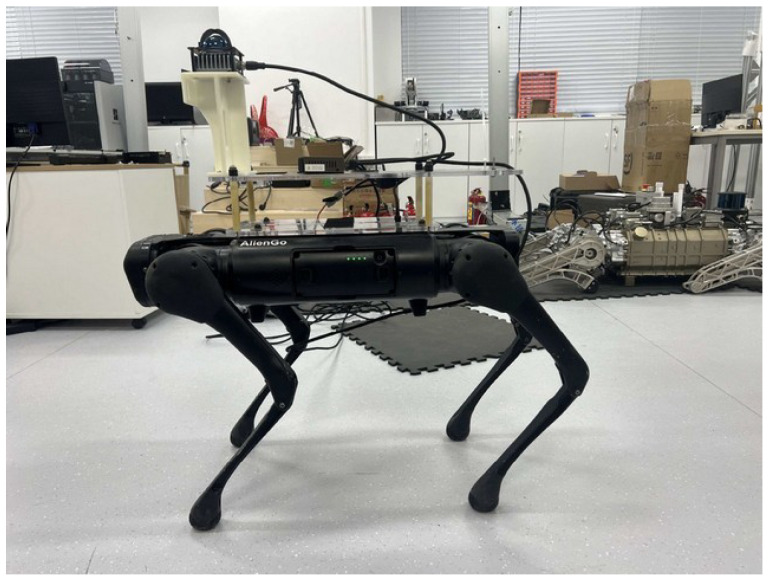
Experimental platform: Unitree Aliengo quadruped robot equipped with Livox Mid-360 LiDAR.

In this study, a Unitree Aliengo quadruped robot is employed as the experimental platform and is equipped with a Livox Mid-360 three-dimensional LiDAR, which provides a horizontal field of view of 360° and a vertical field of view of 59°. The complete platform is shown in Figure 6. The navigation and motion control algorithms are executed on a high-performance laptop computer (Intel Core i9-13900 processor and NVIDIA GeForce RTX 4060 GPU), which communicates with the LiDAR sensor and the robot’s embedded control board via a wired network. All devices are configured within the same IP subnet.

The proposed method primarily relies on the estimation of pedestrians’ positions and velocities. To accurately obtain this information, the PointPillars algorithm is adopted for pedestrian detection [31], and a dedicated pedestrian detection model is trained to meet the experimental requirements. To further enhance the stability and reliability of the detection results, the output bounding boxes are subjected to filtering and post-processing to suppress noise, thereby providing more robust pedestrian state estimates for subsequent navigation decision-making. We quantitatively evaluated the detection and velocity estimation performance of this framework in the indoor corridor experimental scenario. For the main human-robot interaction range of 3–6 m, the model achieves a position root mean square error (RMSE) of 0.078 m and a velocity RMSE of 0.124 m/s; in the short-range interval (<3 m) where collision avoidance decision-making is most sensitive, the position RMSE is 0.042 m and the velocity RMSE is 0.087 m/s, providing reliable state estimation for subsequent TTC calculation and navigation decision-making.

### 5.2. Core Parameter Selection and Sensitivity Analysis

The steering force amplitude coefficient $A_{cp}$ and force decay coefficient $B_{cp}$ (defined in Equations (11) and (12) are the core parameters of the collision-aware orthogonal steering module in the proposed COSFM. They directly determine the robot’s obstacle avoidance response intensity, action trigger timing, navigation efficiency, and social compliance. This section first conducts a single-factor controlled variable experiment to complete the optimization of the two core parameters, and then carries out a systematic sensitivity analysis to verify the robustness and generalizability of the selected parameters.

All parameters of the SFM are fixed to the final settings of this paper (relaxation time τ = 0.5, basic interaction force amplitude *A* = 1.6, effective range coefficient *B* = 1.0). In accordance with Hall’s proxemic theory [32] widely adopted in social navigation research, the minimum human-robot distance of 0.5 m is set as the fundamental evaluation criterion for maintaining pedestrians’ psychological comfort zone and ensuring social compliance with the robot’s navigation behavior across all experiments. When exploring the influence of $A_{cp}$ on system performance, $B_{cp}$ is fixed to the final optimal value of 2.0, and $A_{cp}$ is set to the gradient [1.0, 3.0, 5.0, 7.0, 9.0]. When exploring the influence of $B_{cp}$, $A_{cp}$ is fixed to the final optimal value of 5.0, and $B_{cp}$ is set to the gradient [0.5, 1.0, 2.0, 3.0, 4.0]. The purpose of this experiment is to explore the independent influence of a single parameter on the system behavior, and determine a reasonable candidate interval for each parameter to exclude extreme values with obvious performance degradation that cannot meet the above comfort standard.

The influence of a single parameter on the system behavior and navigation performance is shown in Table 1. For $A_{cp}$: when $A_{cp}$ = 1.0, the steering force is seriously insufficient, resulting in a high collision rate and a navigation success rate of only 10%. When $A_{cp}$ increases to 3.0, the success rate rises to 85%, but the obstacle avoidance response is still delayed, and the minimum interpersonal distance is less than 0.4 m, which cannot guarantee social compliance. When $A_{cp}$ = 5.0, the navigation success rate reaches 100%, the navigation time and path length are optimal, and the minimum interpersonal distance is stably maintained above 0.5 m. When $A_{cp}$ continues to increase to 7.0 and 9.0, the excessive steering force leads to redundant detours, the path length increases by more than 22%, and excessive avoidance even intrudes into the comfort zone of other pedestrians. For $B_{cp}$: when $B_{cp}\leq 1.0$, the force decays too fast, the obstacle avoidance is triggered late, and the robot has abrupt steering and deceleration behaviors, which seriously violate social norms. When $B_{cp}$ = 2.0, the obstacle avoidance trigger timing is reasonable, and all performance indicators reach the optimal level; when $B_{cp}\geq 3.0$, the force decays too slowly, and the robot triggers avoidance for distant pedestrians, resulting in serious path redundancy and a significant reduction in navigation efficiency.

Based on the single-parameter analysis, supplementary experiments are conducted on the cross-combination of $A_{cp}\in \left[3.0,7.0\right]$ and $B_{cp}\in \left[1.0,3.0\right]$. The results show that $A_{cp}$ = 5.0 and $B_{cp}$ = 2.0 achieve the global optimal balance among obstacle avoidance safety, navigation efficiency, and social compliance. This group of parameters is used as the fixed parameter setting for all subsequent simulations and real-world experiments in this paper.

### 5.3. Ablation Study and Multi-Scenario Comparative Validation

To systematically verify the effectiveness of the proposed COSFM method, this section first quantifies the independent contribution of each core innovation module through ablation experiments, to clarify the essential source of the observed performance improvements. Subsequently, the proposed method is compared with two main baseline methods (classical SFM and PTSFM) in four typical human-robot interaction scenarios to comprehensively validate the overall navigation performance of the method.

The selection of baseline methods is justified as follows: (1) The classical SFM [25] is adopted as the fundamental baseline to verify the overall performance improvement of our method over the original SFM framework. (2) PTSFM [14] is selected as the core comparative baseline: it shares the same TTC-based collision prediction framework as our method, with the only core difference being that PTSFM only optimizes the magnitude of interaction forces while our method further reforms the direction of avoidance forces via the OSS and incorporates a quadruped-specific adaptation module.

We also note the ASFM proposed by Ægidius et al. [19], a representative legged robot-oriented SFM variant. However, its two core functional modules (Direction Priority Module and Following Behavior Module) are exclusively designed for first-person vision perception and are fundamentally incompatible with our 360° LiDAR perception framework, which will directly lead to safety risks and navigation failure if retained. To ensure the fairness of the baseline comparison (while maintaining the original algorithm design), we did not include ASFM as a baseline.

To provide a statistically reliable evaluation, each experiment was repeated 20 times under the same experimental settings, and the results are reported as mean ± standard deviation.

#### 5.3.1. Ablation Study

To quantitatively analyze the independent contribution of each core component in the proposed COSFM and to clarify the internal mechanism underlying the method’s performance improvement, we conducted a systematic, controlled ablation experiment in this section. The core objective is to decouple and verify the functional effectiveness of the three key innovations of our proposed COSFM: the TTC, the OSS, and the quadruped-specific adaptation module, so as to determine the core source of the observed performance gains of our method.

The ablation experiment is conducted in the head-on encounter scenario, which is the most representative dynamic human-robot interaction scenario for evaluating obstacle avoidance performance and social compliance. All experimental settings, including the robot’s initial state, target position, pedestrian motion parameters, and environment configuration, are completely consistent with the multi-scenario comparative experiments in the subsequent section. The optimal parameter combination ($A_{cp}=5.0$, $B_{cp}=2.0$) determined in Section 5.2 is uniformly adopted for all groups, and only the target core module is enabled or disabled to strictly follow the single-variable control principle.

We set up five experimental groups with progressive module superposition, and the grouping rules are as follows:

- Group 1 (Baseline): Classical SFM, with all three core modules disabled, to provide the performance baseline;
- Group 2 (Ablation 1): SFM with only the TTC enabled (i.e., the PTSFM baseline used in this paper), to verify the independent contribution of the collision prediction module;
- Group 3 (Ablation 2): SFM with only the OSS enabled, to verify the independent contribution of the lateral avoidance module;
- Group 4 (Ablation 3): SFM with both TTC and OSS enabled, without the quadruped-specific adaptation module, to verify the performance of the core algorithm framework;
- Group 5 (Full Method): The complete proposed COSFM, with all three core modules enabled, to present the full performance of our method.

The evaluation indicators are completely consistent with the full paper, including four core dimensions: navigation success rate (the proportion of reaching the target without collision within the specified time), average navigation time, average path length, and minimum interpersonal distance (the core indicator for evaluating social compliance and pedestrian comfort zone maintenance). The quantitative results of the ablation experiment are shown in Table 2, and the independent contribution of each core module is analyzed in detail as follows:

1. Independent contribution of the TTC. By comparing Group 1 and Group 2, the TTC alone increases the navigation success rate from 10% to 90%, and raises the minimum interpersonal distance from 0.32 m to 0.43 m, which fundamentally solves the serious collision risk of the classical SFM in dynamic interactions, and effectively improves the proactive obstacle avoidance safety of the robot. However, the introduction of TTC alone also leads to an increase in navigation time and path length, resulting in a loss of navigation efficiency.
2. Independent contribution of the OSS. By comparing Group 1 and Group 3, the OSS alone reduces the average navigation time by 13.1% and the average path length by 11.2%, significantly optimizing navigation efficiency but with limited improvement in navigation success rate and social safety distance. Further comparing Group 2 and Group 4, integrating OSS with the TTC mechanism increases the navigation success rate to 100%, reduces the navigation time by 31.6%, and reverses the efficiency loss caused by TTC, while raising the minimum interpersonal distance to 0.49 m, which is close to the social comfort threshold.
3. Independent contribution of the quadruped-specific adaptation module. By comparing Group 4 and Group 5, this module maintains the 100% navigation success rate, further reduces navigation time and path length, and increases the minimum interpersonal distance from 0.49 m to 0.55 m, thereby stably meeting the requirements for the social safety distance. It effectively reduces trajectory fluctuations caused by the gait cycle of quadruped robots, ensures the robot’s dynamic stability during navigation, and is key to the stable deployment of the algorithm on real legged robot platforms and the continuous guarantee of social compliance.

The ablation experiment results clearly demonstrate that all three core modules of the proposed COSFM make irreplaceable contributions to overall performance improvement. The TTC solves the dynamic response delay problem, the OSS improves navigation efficiency and social compliance, and the quadruped-specific adaptation module ensures the stable deployment of the algorithm on the legged robot platform. The complete method, with all modules enabled, achieves an optimal balance between navigation safety, efficiency, and social compliance, clearly clarifying the source of the observed performance gains of our proposed method.

#### 5.3.2. Multi-Scenario Comparative Simulation Experiments

To further verify the comprehensive performance and generalizability of the proposed full COSFM method in diverse human-robot interaction scenarios, we conduct comparative experiments with two mainstream baseline methods: the classical SFM and the PTSFM. Four typical human-robot interaction scenarios are designed for evaluation, and all experimental settings are consistent with the ablation experiment.

Other simulation parameters are set as follows: the maximum linear velocity of the robot $v_{max}$ = 1 m/s, the maximum angular velocity $\omega_{max}$ = 0.3 rad/s, and the maximum acceleration $\alpha_{max}$ = 1.0 m/s^2^. The pedestrian radius $R_{p}$ = 0.4 m. The initial position of the robot is [0, −1], and the initial velocity is 0. The target point is [10, −1], and the robot is considered to have reached the target when the distance between the robot and the target point is less than 0.2 m.

**Figure 7:**
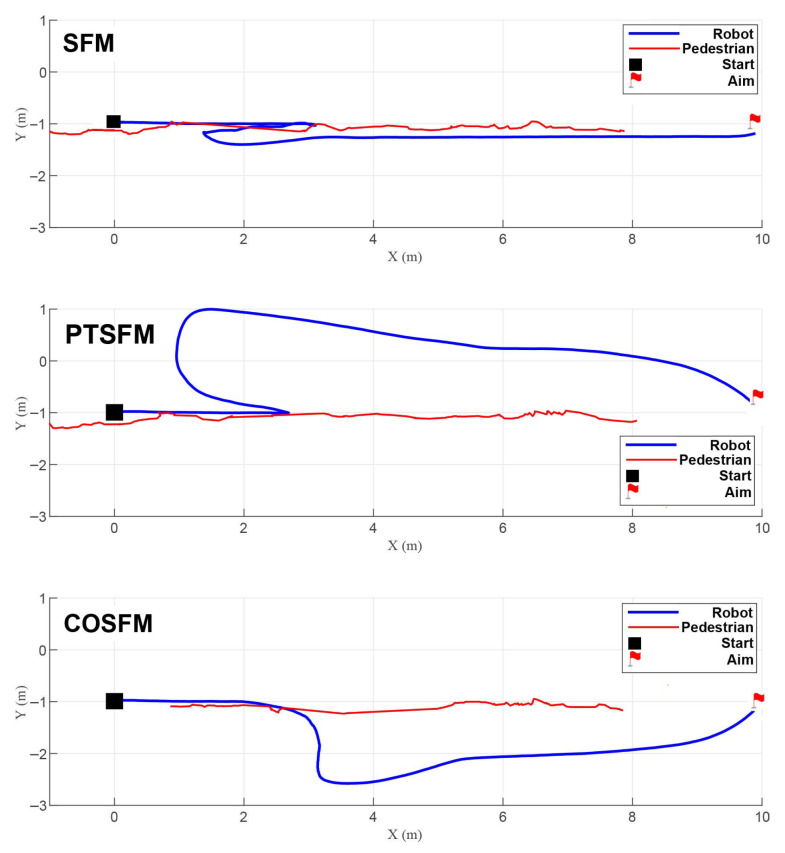
Trajectory comparison in Scenario 1 (head-on encounters).

Scenario 1 (Head-on encounters): As shown in Figure 7, the pedestrian moves head-on at a speed of 1.0 m/s. When facing an oncoming pedestrian, both SFM and PTSFM exhibit clear backward avoidance, resulting in unnatural behavior. Among them, the SFM fails to adjust its posture in time due to the opposite directions of the repulsive force and attractive force, and finally collides; the PTSFM successfully avoids obstacles, but its backward, detouring, and decelerating behaviors lead to a decrease in efficiency. In contrast, the COSFM method proposed in this paper can predict pedestrian movement trends and proactively adopt the appropriate evasion strategy, which aligns with social norms. Its lateral movement mode is also closer to human intuition, effectively ensuring navigation efficiency while maintaining motion continuity.

**Figure 8:**
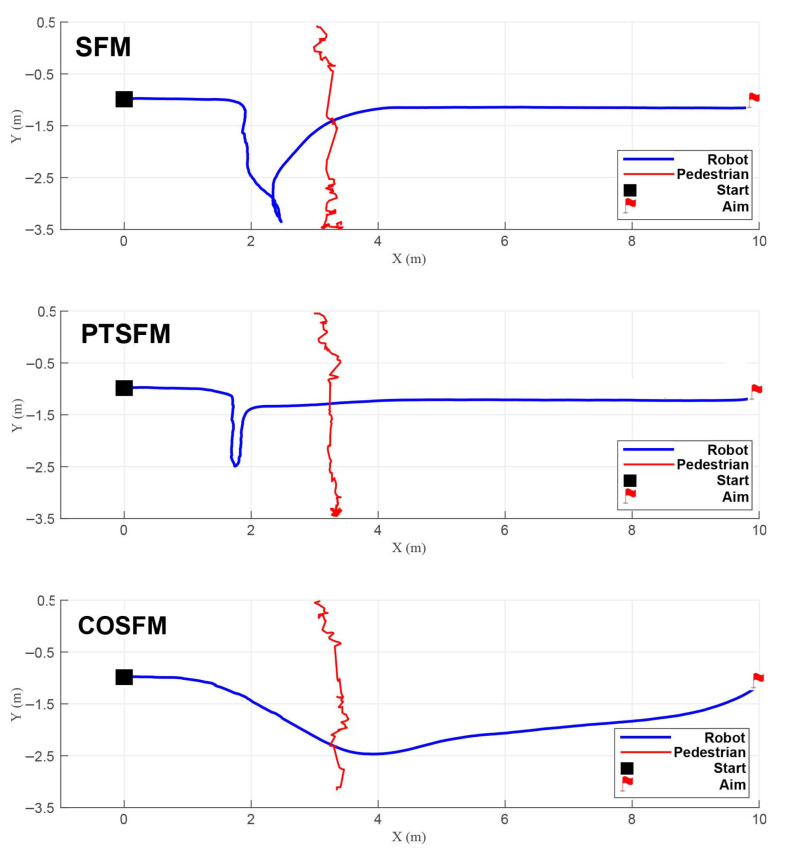
Trajectory comparison in Scenario 2 (intersecting paths).

Scenario 2 (Intersecting paths): As shown in Figure 8, the pedestrian crosses perpendicularly at a speed of 1.0 m/s. At this time, both SFM and PTSFM cause the robot to decelerate unnecessarily when it can pass, and retreat in the direction of the pedestrian’s movement under the action of the repulsive force, finally stagnating and waiting until the pedestrian passes completely before resuming movement. The COSFM proposed in this paper can make flexible decisions and pass first by moderately accelerating, thus avoiding unnecessary waiting and motion loss.

**Figure 9:**
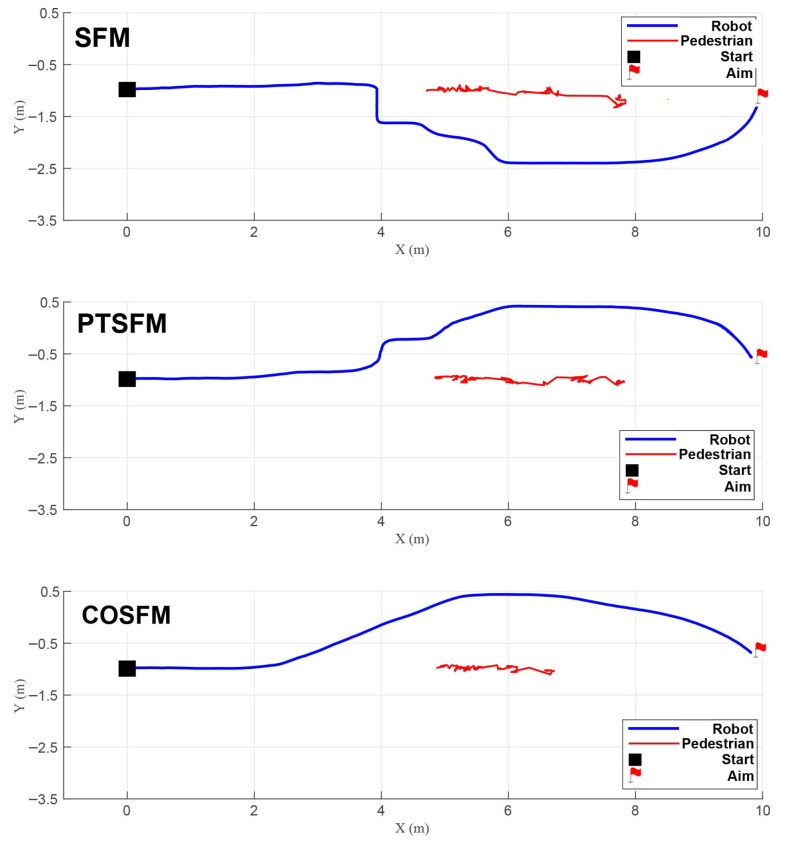
Trajectory comparison in Scenario 3 (active overtaking).

Scenario 3 (Active overtaking): As shown in Figure 9, the pedestrian in front moves slowly at a speed of 0.5 m/s. At this time, all three methods can complete the navigation task, but the SFM and PTSFM produce fluctuations in the motion trajectory due to the influence of the repulsive force during the approach process. The COSFM proposed in this paper shows decisive lateral overtaking behavior with a smooth and coherent trajectory and better navigation fluency.

**Figure 10:**
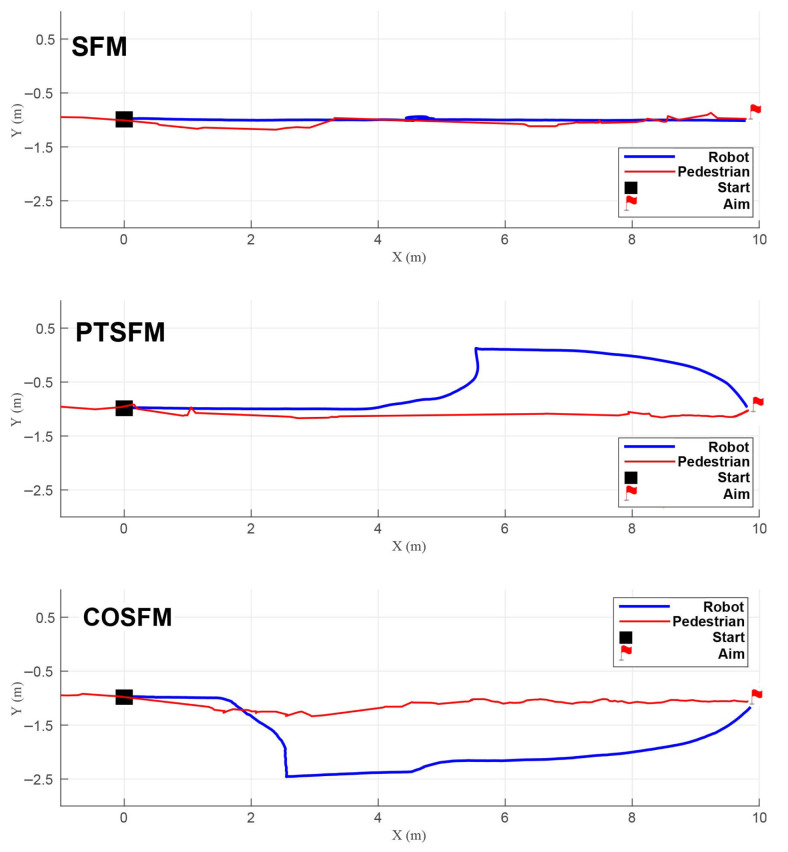
Trajectory comparison in Scenario 4 (passive yielding).

Scenario 4 (Passive yielding): As shown in Figure 10, the pedestrian behind approaches rapidly at a speed of 1.5 m/s. At this time, the SFM fails to respond effectively, leading to a collision; the PTSFM has a delayed response and only makes adjustments after the collision occurs. The COSFM proposed in this paper shows the ability to predict the rear dynamics, can avoid the right in advance, reserve the left overtaking space for pedestrians, and reflect good social adaptability and decision-making foresight.

**Figure 11:**
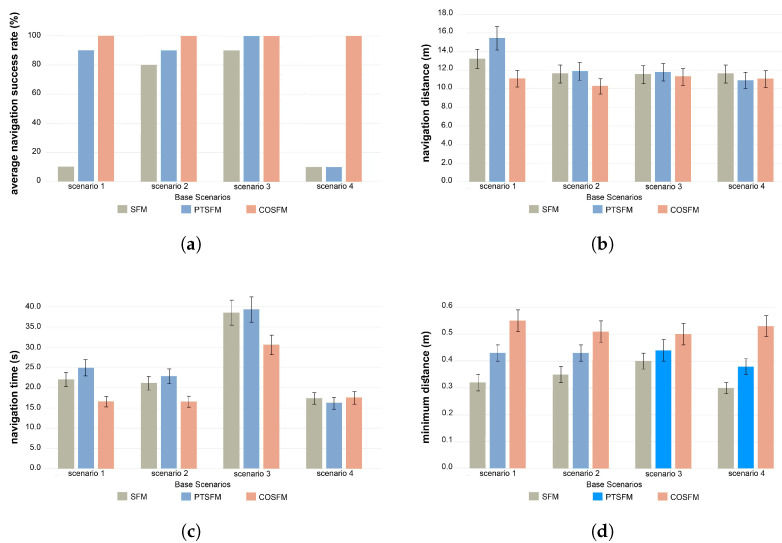
Quantitative performance comparison of three navigation methods in four simulated human–robot interaction scenarios: (**a**) success rate; (**b**) navigation distance; (**c**) navigation time; (**d**) minimum distance.

To quantitatively evaluate the comprehensive performance of the three navigation methods (SFM, PTSFM, and COSFM), 20 groups of independent simulation experiments are further carried out. Four indicators are adopted in the experiments: average navigation success rate (the proportion of reaching the target on time without collision), average navigation distance (actual trajectory length), average navigation time (time consumed from the starting point to the end point), and minimum distance (minimum human-robot distance during navigation). The detailed values of the 20 experimental results for each scenario are summarized in Figure 11 and Table 3. The results show that the COSFM proposed in this paper is superior to the SFM and PTSFM in terms of navigation success rate. The latter two perform poorly at avoiding pedestrian collisions, especially in the passive yielding scenario, with a low success rate. In terms of path length and time consumption, the COSFM shows higher efficiency in the first three scenarios; while in the passive yielding scenario, its average velocity decreases slightly due to the proactive adoption of the avoidance strategy. The minimum distance indicator shows that the COSFM can maintain a safe interval greater than 0.5 m in all scenarios, which aligns with the personal space boundary defined by Hall’s proxemic theory [32], providing a core guarantee for avoiding intrusion into pedestrians’ psychological comfort zones.

### 5.4. Real-World Experiments

To further validate the effectiveness of the proposed COSFM method in real dynamic environments, three real-world navigation scenarios are designed and evaluated on a physical quadruped robot platform. Since the classical SFM exhibits a relatively low navigation success rate across several typical interaction scenarios, the experimental evaluation primarily focuses on comparing PTSFM with the proposed COSFM. The experiments are conducted in a narrow corridor with a width of 3 meters, where the quadruped robot navigates from the start position to a target point located 10 meters ahead. The SFM parameters are set identically to those used in the simulation experiments. Each scenario is independently repeated 20 times.

The evaluation metrics include average navigation success rate, navigation distance, navigation time, and minimum distance. Demonstration videos of the real-world experiments are available at https://b23.tv/myOIWgl (accessed on 15 February 2026).

#### 5.4.1. Navigation Scenarios Setup

**Figure 12:**
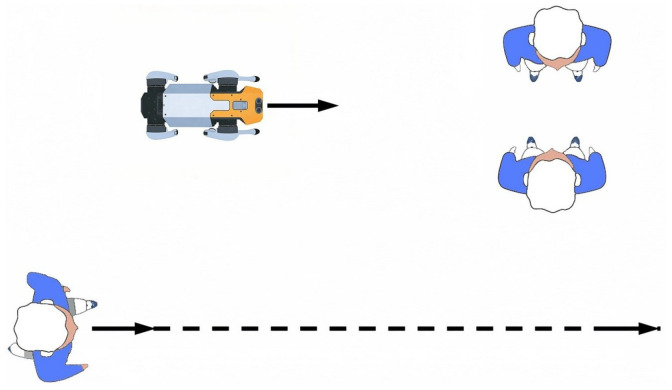
Setup of real-world Scenario 1 (static–dynamic hybrid navigation).

Scenario 1 (Static-dynamic hybrid decision-making): This scenario aims to test the social navigation ability of the quadruped robot in an environment where static obstacles and dynamic pedestrians coexist. Two stationary pedestrians block part of the corridor passage, and one pedestrian crosses horizontally at the same time. The quadruped robot needs to bypass the static crowd through a following strategy on the basis of identifying the dynamic pedestrian’s trajectory, as shown in Figure 12. The experimental results show that the COSFM can prospectively identify static obstacles and pedestrians at the right rear, decelerate in advance and implement the following strategy, thus passing through the blocked area smoothly; while the PTSFM fails to adjust the speed in time, is disturbed during the detour process, and shows motion hesitation and trajectory oscillation, leading to a decrease in traffic efficiency. Statistical results show that under the success criteria set in this paper, the COSFM successfully completes the navigation task in all simulation and experimental scenarios, and its efficient following strategy is superior to the PTSFM in terms of average navigation distance and time. The latter causes an average increase of about 12% in navigation time due to behavioral hesitation.

**Figure 13:**
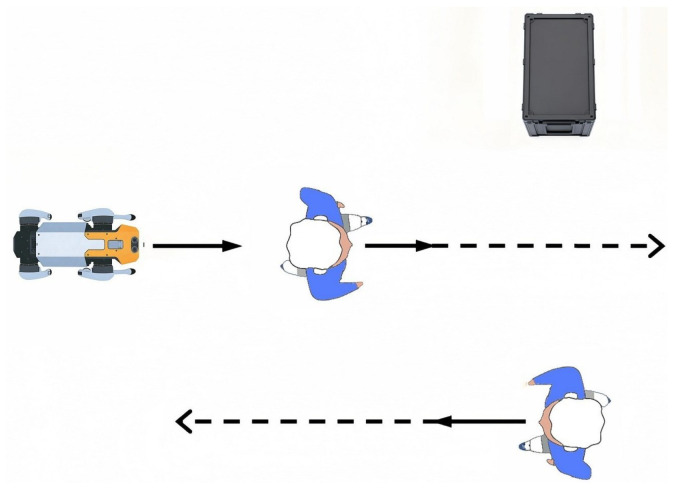
Setup of real-world Scenario 2 (sudden obstacle avoidance).

Scenario 2 (Sudden obstacle avoidance decision-making): This scenario is used to evaluate the real-time adaptability of the navigation algorithm under sudden interference. The setup includes a pedestrian moving forward at a low speed and a pedestrian crossing laterally, and the quadruped robot needs to overtake the former while avoiding the sudden movement of the latter, as shown in Figure 13. The experiment shows that the COSFM can accelerate to overtake on the premise of maintaining the priority of the pedestrian in front to pass, and predict the crossing pedestrian to detour without requiring pedestrians to give way throughout the process; the PTSFM can complete the detour, but has a delayed decision response, and the robot pauses and sways many times during the navigation process and has to wait for the pedestrian to pass before continuing to move. The corresponding data show that the average navigation time of the COSFM is about 37% shorter than that of the PTSFM.

**Figure 14:**
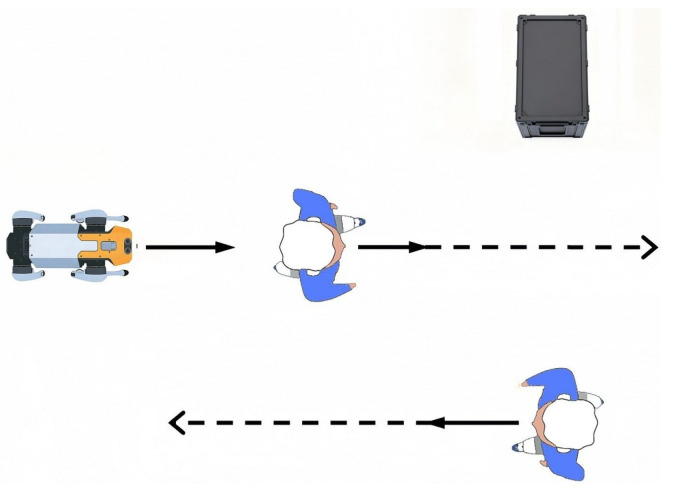
Setup of real-world Scenario 3 (multi-directional pedestrian flow navigation).

Scenario 3 (Multi-directional pedestrian flow decision-making): This scenario aims to test the robot’s dynamic path selection ability in a multi-constraint space. The scenario is equipped with an oncoming pedestrian, a pedestrian moving in the same direction, and a left obstacle, as shown in Figure 14. The experimental results show that the COSFM makes reasonable decisions through intention prediction: it first keeps following, and then accelerates to cross from the right after the oncoming pedestrian passes; the PTSFM shows direction uncertainty and often chooses to detour to the left, but often falls into local stagnation due to obstacles and pedestrian blocking. Therefore, the COSFM is superior to the PTSFM in terms of path planning stability and average moving speed. The latter causes fluctuations in navigation success rate and longer average navigation time due to invalid path selection.

All real-world experiments were conducted in a 3-meter-wide indoor corridor. Pedestrians followed predefined trajectories and maintained constant speeds for each scenario, ensuring consistent and repeatable dynamic interaction conditions across all trials. The pedestrian state estimator and upper-layer COSFM navigation decision module run at 10 Hz, fully synchronized with the navigation planning cycle; the lower-layer NMPC + WBC motion control module runs at 500 Hz for high-frequency trajectory tracking and dynamic balance control. This standard hierarchical control architecture ensures full functional compatibility: the 10 Hz update rate is fully sufficient to capture the slow dynamic motion of walking pedestrians, while the 500 Hz high-frequency MPC guarantees stable tracking of the upper-layer reference trajectory. The effective detection range of the Livox Mid-360 LiDAR for pedestrian targets was configured to 0.5–6 m, balancing real-time perception efficiency and navigation decision foresight. A dedicated safety operator monitored the robot throughout all experiments using a handheld emergency stop remote controller and a full manual override function, ensuring the safety of personnel and the robot platform.

#### 5.4.2. Result Analysis

**Figure 15:**
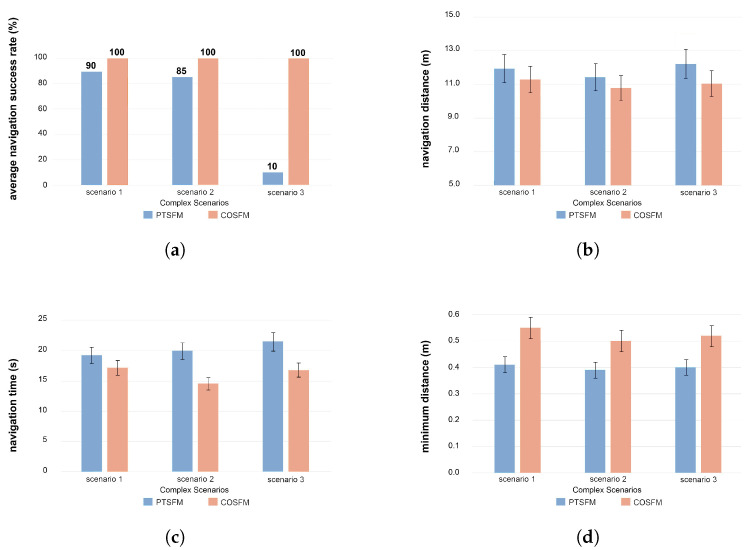
Quantitative performance comparison of two navigation methods in three real-world human–robot interaction scenarios: (**a**) success rate; (**b**) navigation distance; (**c**) navigation time; (**d**) minimum distance.

In terms of navigation success rate, as shown in Figure 15a, the proposed COSFM achieves a 100% navigation success rate in all three real-world dynamic scenarios, while the success rate of the PTSFM baseline decreases significantly with the increase of scene complexity, dropping to only 10% in the multi-directional pedestrian flow scenario. This result fully demonstrates that the TTC-based collision prediction mechanism and the rule-driven orthogonal steering decision-making framework of COSFM can effectively improve the proactive obstacle avoidance capability and decision-making robustness of the quadruped robot in unstructured real dynamic environments, fundamentally avoiding the problems of delayed response and local stagnation in the traditional predictive SFM method.

For navigation efficiency, the statistical results in Table 4 and Figure 15b–d show that COSFM achieves significant optimization in both navigation time and path length compared with PTSFM. Specifically, COSFM shortens the navigation time by 10.7% in the static-dynamic hybrid scenario, by 27.1% in the sudden obstacle avoidance scenario, and by 22.0% in the multi-directional pedestrian flow scenario, with an average path length reduction of 6.9% across all scenarios. Meanwhile, the average navigation speed of COSFM is consistently higher than that of PTSFM in all scenarios, which benefits from the lateral priority avoidance strategy of COSFM: it avoids the frequent deceleration, stagnation and backward retreat behaviors of traditional methods, and maintains the continuity and fluency of the robot’s motion through orthogonal steering detour, which is also consistent with the simulation experimental conclusions.

In terms of social compliance, the minimum interpersonal distance indicator in Table 4 shows that COSFM can consistently maintain a minimum interpersonal distance of more than 0.5 m across all real-world scenarios, which aligns with the personal space boundary defined by Hall’s proxemic theory. In contrast, the minimum interpersonal distance of PTSFM is below 0.45 m in all scenarios, posing a high risk of intruding into pedestrians’ psychological comfort zones. This result verifies that the social-norm fusion design of COSFM can effectively protect pedestrians’ personal space while performing obstacle avoidance and significantly improve the social acceptability of the quadruped robot’s navigation behavior in human-robot shared environments.

**Figure 16:**
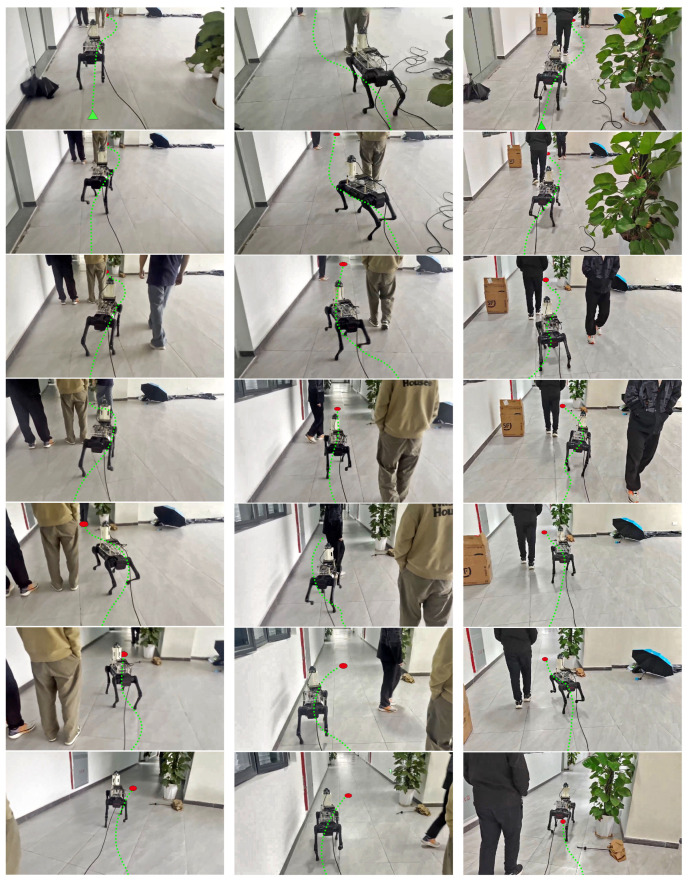
Snapshots of three complex scenarios real-world experiments.

In summary, the physical experiment results show that by combining the collision prediction mechanism with social rules, the COSFM improves efficiency and reliability in dynamic human-robot mixed environments while ensuring navigation safety. Its core advantages are reflected in prospective behavior-based decision-making and a high degree of social compliance, providing key technical support for the deployment of quadruped robots in complex real-world scenarios. To further evaluate whether the performance improvements of COSFM over PTSFM are statistically significant, we conducted Wilcoxon signed-rank tests across all scenarios. The resulting p-values and significance levels are reported in Table 5. The statistical analysis in Table 5 shows that most performance improvements achieved by COSFM are statistically significant, particularly in navigation time and minimum human–robot distance. Snapshots of real-world social experiments are shown in Figure 16.

To verify the impact of detection uncertainty on TTC computation and minimum-distance metrics, we conducted supplementary sensitivity experiments: we injected velocity noise (matching the measured RMSE of 0.087 m/s) into short-range pedestrian state estimates and re-ran 10 sets of real-world trials. Results show that the relative error of TTC computation remains below 10%, and the minimum human-robot distance only fluctuates within ±0.03 m (still stably ≥ 0.47 m, above the social comfort threshold). The gait-aware TTC offset design and Kalman filter-based state smoothing effectively suppress noise propagation, ensuring navigation decisions remain robust even under perception anomalies.

Benefiting from the lateral velocity limitation introduced in Section 4.1.1, the ZMP clipping mechanism was activated in only 3.2% of real-world navigation frames, and no ZMP violations were observed throughout all experiments. This confirms that the combined lateral motion restriction and ZMP constraint effectively maintain dynamic stability under the social navigation scenarios studied.

Although the effectiveness of the proposed COSFM algorithm has been validated across multiple typical human-robot interaction scenarios, current real-world experiments are mainly conducted in controlled indoor environments, such as corridors and laboratories, where pedestrian densities are lower than in open public spaces. To further assess the generality and robustness of the proposed approach, future work will focus on deploying the algorithm in more complex and unstructured public environments, such as large shopping malls and campus plazas, and on introducing higher-density, more stochastic pedestrian flows for stress testing. In addition, in subsequent research, we will further conduct perceptual user studies with actual human participants to quantitatively verify pedestrians’ subjective comfort and acceptance of the robot’s navigation behavior.

## 6. Conclusions

To address the key limitations of existing social force models in dynamic pedestrian avoidance for quadruped robots, including delayed dynamic response, insufficient consideration of pedestrians’ comfort zones, and poor adaptability to legged platforms, this paper proposes a collision-aware orthogonal steering social force model that integrates collision prediction and social norms, with systematic optimization for quadruped robot locomotion characteristics.

Compared with mainstream existing research, the core novelties of this work are threefold: first, it unifies the TTC and socially compliant OSS within the SFM framework, reshaping the obstacle avoidance logic from deceleration-dominated to lateral steering-prioritized behavior; second, it systematically integrates quadruped-specific gait awareness, leg safety space, and dynamic balance constraints, bridging the gap between social force modeling and stable deployment on legged robots; third, it designs a rule-driven steering decision mechanism for four typical human-robot interaction scenarios, significantly improving the social compliance of navigation behaviors.

Compared with representative DRL methods CADRL and SA-CADRL, our COSFM achieves 100% navigation success rate in both simulation and real-world tests, stably maintains the 0.5 m social comfort distance, and delivers full interpretability and zero-fine-tuning sim-to-real transfer, showing significant advantages for practical safety- critical deployment.

Both simulation and real-world experiments demonstrate that the proposed method comprehensively outperforms classical SFM and PTSFM baselines in dynamic human-robot shared environments, which is the core application scenario of this work. While its performance is comparable to mature path-planning algorithms in non-core scenarios such as pure static obstacle navigation, its core advantage lies in simultaneously optimizing navigation safety, motion efficiency, social compliance, and dynamic stability for quadruped robot social navigation. Meanwhile, the rule-based model has clear interpretability and excellent sim-to-real transfer performance, which greatly reduces the difficulty of engineering deployment.

Future work will optimize the method for high-density multi-pedestrian scenarios and conduct systematic user studies to further verify and improve the social acceptability of the robot’s navigation behavior.

## Figures and Tables

**Table 1:** Results of single-factor sensitivity pre-experiment.

Parameter Group	Success Rate (%)	Nav. Time (s)	Path Length (m)	Min. Distance (m)
$A_{cp}$ = 1.0	10	22.03	13.22	0.32
$A_{cp}$ = 3.0	85	18.62	12.08	0.41
$A_{cp}$ = 5.0	100	16.57	11.10	0.55
$A_{cp}$ = 7.0	100	19.34	13.57	0.69
$A_{cp}$ = 9.0	100	21.76	15.02	0.78
$B_{cp}$ = 0.5	15	21.47	12.85	0.34
$B_{cp}$ = 1.0	90	17.83	11.64	0.43
$B_{cp}$ = 2.0	100	16.57	11.10	0.55
$B_{cp}$ = 3.0	100	18.91	12.76	0.63
$B_{cp}$ = 4.0	100	20.82	14.13	0.72

**Table 2:** Quantitative results of the ablation experiment (mean ± std over 20 trials).

Experimental Group	Success Rate (%)	Nav. Time (s)	Path Length (m)	Min. Distance (m)
Group 1: Classical SFM	10%	22.03 ± 1.54	13.22 ± 0.92	0.32 ± 0.03
Group 2: SFM + TTC (PTSFM)	90%	24.92 ± 1.74	15.45 ± 1.08	0.43 ± 0.03
Group 3: SFM + OSS	45%	19.15 ± 1.34	11.74 ± 0.82	0.39 ± 0.03
Group 4: SFM + TTC + OSS	100%	17.05 ± 1.19	11.41 ± 0.80	0.49 ± 0.04
Group 5: Full COSFM (Ours)	100%	16.57 ± 1.16	11.10 ± 0.78	0.55 ± 0.04

**Table 3:** Statistical performance comparison in four simulated human–robot interaction scenarios (mean ± std over 20 trials).

Scenario	Method	Success Rate (%)	Nav. Time (s)	Path Length (m)	Min. Distance (m)
Scenario 1	Classical SFM	10	22.03 ± 1.76	13.22 ± 1.06	0.32 ± 0.03
	PTSFM	90	24.92 ± 1.99	15.45 ± 1.24	0.43 ± 0.03
	COSFM (Ours)	100	16.57 ± 1.33	11.10 ± 0.89	0.55 ± 0.04
Scenario 2	Classical SFM	80	21.16 ± 1.69	11.64 ± 0.93	0.35 ± 0.03
	PTSFM	90	22.85 ± 1.83	11.88 ± 0.95	0.43 ± 0.03
	COSFM (Ours)	100	16.58 ± 1.33	10.28 ± 0.82	0.51 ± 0.04
Scenario 3	Classical SFM	90	38.53 ± 3.08	11.56 ± 0.92	0.40 ± 0.03
	PTSFM	100	39.37 ± 3.15	11.81 ± 0.94	0.44 ± 0.04
	COSFM (Ours)	100	30.62 ± 2.45	11.33 ± 0.91	0.50 ± 0.04
Scenario 4	Classical SFM	10	17.36 ± 1.39	11.63 ± 0.93	0.30 ± 0.02
	PTSFM	10	16.28 ± 1.30	10.91 ± 0.87	0.38 ± 0.03
	COSFM (Ours)	100	17.57 ± 1.41	11.07 ± 0.89	0.53 ± 0.04

**Table 4:** Statistical performance comparison in three real-world navigation scenarios (mean ± std over 20 trials).

Scenario	Method	Success Rate (%)	Nav. Time (s)	Path Length (m)	Min. Distance (m)
Scenario 1	PTSFM	90	19.29 ± 1.35	11.96 ± 0.84	0.41 ± 0.03
	COSFM (Ours)	100	17.22 ± 1.21	11.32 ± 0.79	0.55 ± 0.04
Scenario 2	PTSFM	85	20.03 ± 1.40	11.46 ± 0.80	0.39 ± 0.03
	COSFM (Ours)	100	14.60 ± 1.02	10.80 ± 0.76	0.50 ± 0.04
Scenario 3	PTSFM	10	21.56 ± 1.51	12.25 ± 0.86	0.40 ± 0.03
	COSFM (Ours)	100	16.81 ± 1.18	11.07 ± 0.77	0.52 ± 0.04

**Table 5:** Statistical significance analysis between COSFM and PTSFM across all scenarios (* p<0.05, ** p<0.01, *** p<0.001, n.s.: not significant).

Metric	Scenario	*p*-Value	Significance
Navigation Time	Sim-1	0.0018	**
	Sim-2	0.0026	**
	Sim-3	<0.001	***
	Sim-4	0.21	n.s.
	Real-1	0.004	**
	Real-2	0.006	**
	Real-3	0.002	**
Path Length	Sim-1	0.012	*
	Sim-2	0.008	**
	Sim-3	0.001	***
	Sim-4	0.37	n.s.
	Real-1	0.041	*
	Real-2	0.018	*
	Real-3	0.009	**
Min. Distance	Sim-1	<0.001	***
	Sim-2	<0.001	***
	Sim-3	0.002	**
	Sim-4	<0.001	***
	Real-1	<0.001	***
	Real-2	<0.001	***
	Real-3	<0.001	***

## Data Availability

The original contributions presented in the study are included in the article, and further inquiries can be directed to the corresponding author.
